# Demographic histories and genetic diversities of Fennoscandian marine and landlocked ringed seal subspecies

**DOI:** 10.1002/ece3.1193

**Published:** 2014-08-19

**Authors:** Tommi Nyman, Mia Valtonen, Jouni Aspi, Minna Ruokonen, Mervi Kunnasranta, Jukka U Palo

**Affiliations:** 1 Department of Biology, University of Eastern FinlandPO Box 111, Joensuu, FI-80101, Finland; 2 Institute for Systematic Botany, University of Zurich, Zollikerstrasse 107Zurich, CH-8008, Switzerland; 3 Department of Biology, University of OuluPO Box 3000, Oulu, FI-90014, Finland; 4 Laboratory of Forensic Biology, Hjelt Institute, University of HelsinkiPO Box 40, Helsinki, FI-00014, Finland

**Keywords:** Approximate Bayesian computing, bottleneck, demographic history, founder event, genetic diversity, island populations

## Abstract

Island populations are on average smaller, genetically less diverse, and at a higher risk to go extinct than mainland populations. Low genetic diversity may elevate extinction probability, but the genetic component of the risk can be affected by the mode of diversity loss, which, in turn, is connected to the demographic history of the population. Here, we examined the history of genetic erosion in three Fennoscandian ringed seal subspecies, of which one inhabits the Baltic Sea ‘mainland’ and two the ‘aquatic islands’ composed of Lake Saimaa in Finland and Lake Ladoga in Russia. Both lakes were colonized by marine seals after their formation *c*. 9500 years ago, but Lake Ladoga is larger and more contiguous than Lake Saimaa. All three populations suffered dramatic declines during the 20th century, but the bottleneck was particularly severe in Lake Saimaa. Data from 17 microsatellite loci and mitochondrial control-region sequences show that Saimaa ringed seals have lost most of the genetic diversity present in their Baltic ancestors, while the Ladoga population has experienced only minor reductions. Using Approximate Bayesian computing analyses, we show that the genetic uniformity of the Saimaa subspecies derives from an extended founder event and subsequent slow erosion, rather than from the recent bottleneck. This suggests that the population has persisted for nearly 10,000 years despite having low genetic variation. The relatively high diversity of the Ladoga population appears to result from a high number of initial colonizers and a high post-colonization population size, but possibly also by a shorter isolation period and/or occasional gene flow from the Baltic Sea.

## Introduction

Island populations have higher rates of extinction than mainland ones, mainly because they tend to be small and, hence, more vulnerable to environmental and demographic stochasticity (Ricklefs 2010; Wootton and Pfister 2013). Small population size also has inexorable genetic consequences – loss of neutral and adaptive variation and increase of inbreeding – that can hasten extinction by reducing individual fitness (Frankham 1998; O'Grady et al. 2006). However, the adversity of genetic effects varies from species to species (Jamieson 2007), and may also depend on the rate at which diversity is lost; in particular, it has been hypothesized that gradual erosion of variation allows purging of detrimental alleles, which would lessen inbreeding depression (Crnokrak and Barrett 2002; Bouzat 2010). However, experimental evidence and investigations of captive populations have cast doubt on the existence or effectiveness of purging (Boakes et al. 2007; Leberg and Firmin 2008).

**Figure 1:**
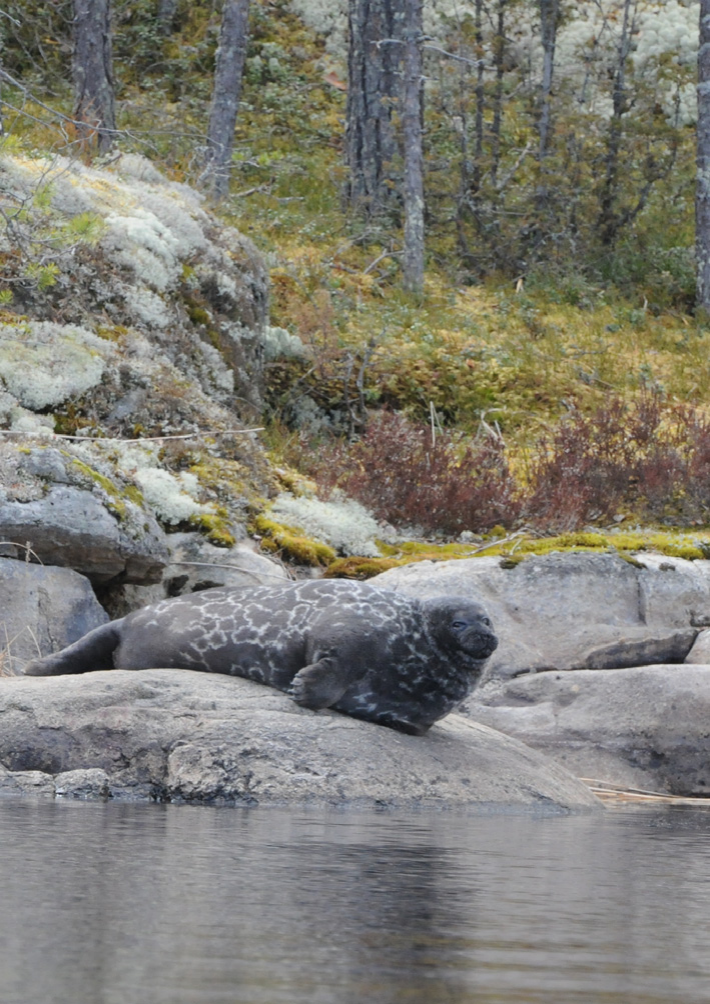
Adult Saimaa ringed seal (*Phoca hispida saimensis*) hauling out on lakeside rocks. Photograph by Mervi Kunnasranta.

**Figure 2:**
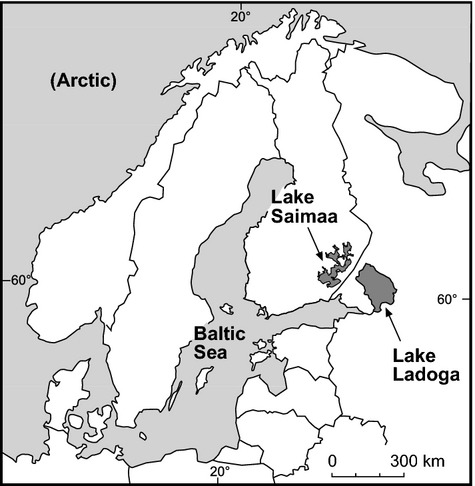
Map showing the distributions of the marine and landlocked Fennoscandian ringed seal subspecies.

Assessing standing genetic diversity is routine, but determining the rate of diversity loss is far more challenging, as this requires knowledge on the initial levels of variation as well as on isolation times (Wayne et al. 1991; Wang et al. 2014). Perhaps surprisingly, a promising model system for studying insular genetic erosion is provided by ringed seals (*Phoca hispida*) that inhabit the large Fennoscandian lakes Saimaa and Ladoga (Figs.1, 2). From the perspective of seals, these lakes represent ‘islands’ separated from the marine ‘mainland’ and, importantly, the initial diversity of the landlocked populations can be estimated on the basis of extant marine seals. In addition, the ages of the lakes are well known, as they owe their origin to geological events that were set in motion *c*. 10,000 years ago when the last glacial period was coming to an end. As the Scandinavian ice shield retreated northwest, isostatic land uplift in the pre-Baltic Yoldia Sea area led to the formation of numerous lakes in which ringed seals could be trapped (Eronen et al. 2001). Of these – possibly numerous – lacustrine populations, only two survive to this day: the Saimaa ringed seal (*P. h. saimensis*) in southeastern Finland, and the Ladoga ringed seal (*P. h. ladogensis*) in northwestern Russia (Ukkonen 2002; Saarnisto 2011). During their extended isolation, these populations have evolved into distinct subspecies that are morphologically, behaviorally, and genetically differentiated from each other, as well as from the direct descendants of their Yoldian ancestors in the current Baltic Sea (*P. h. botnica*) (Sipilä et al. 1996; Hyvärinen et al. 1999; Amano et al. 2002; Berta and Churchill 2012; Valtonen et al. 2012).

Previous assessments based on nuclear and mitochondrial markers have shown that the Saimaa subspecies is genetically depauperate compared to its Fennoscandian sister populations (Palo et al. 2003; Valtonen et al. 2012). However, the trajectory of the loss remains uncertain, partly because all Fennoscandian seal populations underwent dramatic human-caused declines during the 20th century (Kokko et al. 1999). The recent bottleneck was particularly severe in Lake Saimaa, in which the number of seals fell below 200 in the mid-1980s (Hyvärinen et al. 1999; Sipilä 2003). The population has since slowly recovered to slightly over 300 individuals (Metsähallitus Natural Heritage Services 2013), but it remains critically endangered. Reductions were equally dramatic in the Baltic and Ladoga populations, but their head counts remained in the thousands (currently *c*. 15,000 and >5000, respectively; Sipilä et al. 1996; Sundqvist et al. 2012; Trukhanova et al. 2013), which could maintain genetic variation at or near pre-bottleneck levels.

In this study, we aimed at estimating genetic diversity in, and differentiation among, Fennoscandian ringed seal populations, with a specific focus on the demographic histories of the two landlocked subspecies. In particular, we wanted to find out whether the genetic uniformity of the Saimaa subspecies is a legacy of gradual diversity erosion during its long isolation, or a result of more abrupt losses during the colonization of the lake (i.e., a founder effect) and/or the recent bottleneck. On this account, we combined new data from 17 microsatellite loci (Valtonen et al. 2014) with previously published mitochondrial control-region sequence data (Valtonen et al. 2012) to estimate the divergence times and past effective population sizes of the three focal subspecies. Specifically, we took advantage of the ability of recently developed approximate Bayesian computation methods to evaluate the fit of alternative, increasingly complex hypotheses, which assume either constant postglacial sizes in all populations, or invoke an assumption of bottlenecks at colonization and/or during the last decades.

## Methods

### Data acquisition

For Saimaa and Ladoga seals, we used samples from a tissue bank maintained by the University of Eastern Finland and the Natural Heritage Services of Metsähallitus, while Baltic individuals were provided by the Finnish Game and Fisheries Research Institute. Microsatellite variation was inferred at 17 variable loci; detailed methods and data characteristics are described in Valtonen et al. (2014). The possibility of genotyping errors was investigated by analyzing the data in MICRO-CHECKER v. 2.2.3 (van Oosterhout et al. 2004) using Bonferroni-adjusted 95% confidence intervals, and null allele frequencies at each locus for each population were estimated using Chapuis and Estoup's (2007) FREENA software. The microsatellite data were supplemented with 707 bp of mitochondrial control-region sequences from the same individuals collected by Valtonen et al. (2012). For the Saimaa subspecies, microsatellite genotypes were obtained for 172 individuals (=*N*_ms_), and mtDNA sequences for 166 of these (=*N*_mt_). For the Ladoga subspecies *N*_ms_ = *N*_mt_ = 16, and for the Baltic population *N*_ms_ = 21 and *N*_mt_ = 19.

### Estimation of genetic diversity and differentiation

We used ARLEQUIN v. 3.5.1.2 (Excoffier and Lischer 2010) to estimate basic genetic diversity measures [number of alleles (*A*), and observed (*H*_O_) and expected (*H*_E_) heterozygosities] as well as Wright's in breeding coefficient (*F*_IS_) for each subspecies. HP-RARE v. 1.1 (Kalinowski 2004) was used to estimate allelic richness (*A*_R_) by rarefaction to the smallest sample size (*N* = 16). Deviations from Hardy–Weinberg equilibrium (HWE) in individual loci, as well as linkage disequilibrium across locus pairs, were evaluated in ARLEQUIN. The overall loss of genetic diversity over time in the landlocked populations was estimated as *F*_*e*_ = 1 – (*H*_lake_/*H*_Baltic_), assuming that the (observed) heterozygosity in the Baltic population represents the level at the start of the lacustrine isolation (*cf*. Palo et al. 2003).

To investigate the amount of genetic differentiation among the subspecies, we performed AMOVA (Excoffier et al. 1992) and estimated pairwise *F*_ST_ values in ARLEQUIN. Significance levels were determined by 10,000 permutations. Because significant deviations from HWE were observed in the loci *Hg1.4* and *Hg3.6* in all populations, and because the estimated null-allele frequencies in these loci were relatively high (*r* ≥ 0.18) in the Baltic and Ladoga samples (see Results), global and pairwise *F*_ST_ values were also calculated while correcting for the presence of null alleles in these loci in FREENA.

Genetic differences among individuals originating from the three populations were visualized with factorial correspondence analysis (FCA) implemented in the program GENETIX v. 4.05.2 (Belkhir et al. 2004). Distinctness of the subspecies was further studied using Bayesian genotype assignment analyses in STRUCTURE v. 2.3.4 (Pritchard et al. 2000; Hubisz et al. 2009), while excluding prior information on the sampling population. We used the admixture model with correlated allele frequencies, and tested the number of clusters (*K*) from 1 to 10 using a burn-in period of 100,000 followed by 500,000 MCMC iterations, with 20 replicate analyses per each value of *K* to ensure convergence. The analysis was repeated using the no-admixture model with otherwise similar settings. In both cases, we used the ad hoc approach of Evanno et al. (2005), implemented in STRUCTURE HARVESTER v. 0.6.93 (Earl and vonHoldt 2011), to determine the number of clusters that best fits the data.

### ABC analyses

**Figure 3:**
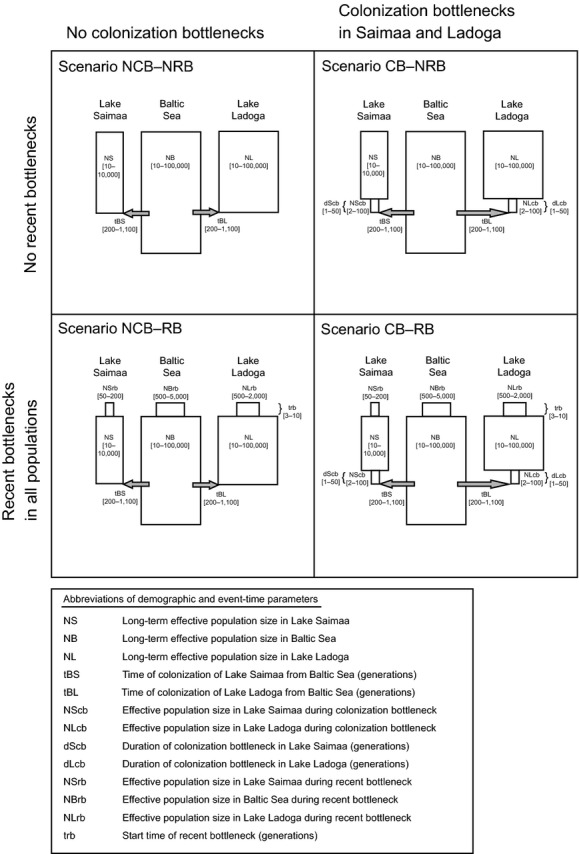
Schematic illustration of the four hypothetical scenarios evaluated using approximate Bayesian computing (ABC), with the uniform prior ranges used for demographic and event-time parameters given in square brackets. Note that relative times and population sizes are not to scale.

Before approximate Bayesian computing (ABC) analyses in DIYABC v. 1.0.4.46 (Cornuet et al. 2008, 2010), we defined four incrementally complex hypotheses on the colonization and population histories of lakes Saimaa and Ladoga (Fig.3). All scenarios assumed that the two lakes were colonized independently from a Baltic source population. Scenario ‘No Colonization Bottleneck – No Recent Bottleneck’ (NCB–NRB) assumed constant population sizes through time in all populations, Scenario CB–NRB included independent initial colonization bottlenecks in the landlocked populations, Scenario NCB–RB assumed only a recent bottleneck starting in all populations during the 20th century, and Scenario CB–RB involved both colonization bottlenecks and a marked recent reduction in population size in all subspecies.

Broad uniform priors for current effective population sizes (Fig.3) were set based on published estimates (Sipilä et al. 1996; Sipilä 2003; Sundqvist et al. 2012; Metsähallitus Natural Heritage Services 2013; Trukhanova et al. 2013) and preliminary test runs. The independent U[200–1100] generations priors for the timing of both colonization events correspond to 2200–12,100 years assuming an 11-year generation time for ringed seals, estimated from the age-specific fecundity scheme in Smith (1973; see Palo et al. 2001). This range should encompass the origination times of both lakes (Eronen et al. 2001; Saarnisto 2011), and the lower limit is close enough to the Recent to take into account the possibility of a wide connection between Lake Ladoga and the Baltic Sea until *c*. 4000 years ago (see below). For the hypothesized colonization bottlenecks, we set the effective population size prior as U[2–100] and the duration prior to U[1–50] generations in both lakes, reflecting the common assumption that colonization bottlenecks are relatively brief (e.g., Keller et al. 2012). The temporal prior of the recent bottleneck was set to U[3–10] generations, which, given the aforementioned generation time, corresponds to a sharp population decline commencing between 1903 and 1980 in all subspecies (*cf*. Sipilä et al. 1996; Kokko et al. 1999; Sipilä 2003; Sundqvist et al. 2012).

For the microsatellite loci, we employed a generalized stepwise mutation model (Estoup et al. 2002) with a maximum of 40 contiguous dinucleotide allelic states allowed for each locus. Default prior ranges were used for all parameters, with the exception of the lower bound of the mean mutation rate, which was lowered to 10^−5^, and the corresponding value of the individual locus mutation rate, which was set to 10^−6^ (Table S1 in Appendix S1). These changes were made on the basis of exploratory runs showing that posterior distributions of mutation-rate parameters tended to fit poorly within the default prior bounds (see also Fontaine et al. 2012). For mitochondrial control-region sequences, we used a HKY+I+G model of substitution, with the proportion of invariant sites set to 88% and the gamma shape parameter to 1.40 (see Valtonen et al. 2012). For the transition/transversion rate ratio (kappa), we used default prior limits, but the upper bound of the prior on the mutation rate (per site and generation) was raised to 10^−5^ (Table S1 in Appendix S1). As above, this change was based on preliminary test runs, but an elevated upper limit also conforms with control-region mutation rates estimated by Valtonen et al. (2012).

For measuring the fit of simulated datasets to the observed data, we selected three single-sample summary statistics (mean number of alleles, expected heterozygosity, and mean Garza–Williamson's *M*) and three two-sample statistics (mean number of alleles, *F*_ST_, and classification index) for microsatellites. For mitochondrial DNA sequences, we used four single-sample targets (numbers of haplotypes and segregating sites, and mean and variance of pairwise differences) and three two-sample statistics (numbers of haplotypes and segregating sites, and *F*_ST_). Following the recommendation of Cornuet et al. (2010), subsequent model checking was based on a separate set of summary statistics (Table S2 in Appendix S1).

A total of four million simulated datasets were generated using the Vuori cluster of the Center for Scientific Computing in Espoo, Finland. This corresponds to roughly one million simulations per scenario, given that the prior probability of scenarios was set to equal. Posterior support for each scenario was assessed based on (1) a direct estimate, in which 500 datasets with summary statistics closest to the target values were extracted from the simulated pool, and (2) a polychotomous logistic regression approach (Cornuet et al. 2008) based on the best-fitting 1% (40,000) of simulated datasets. Posterior distributions of parameters according to individual scenarios were estimated by local linear regression on *logit*-transformed parameter values on the basis of 0.1% (1000) of best-fitting simulated datasets.

Because ABC inferences theoretically can be influenced by the presence of null alleles, the analyses described above were repeated with a dataset from which we excluded the two microsatellite loci (*Hg1.4* and *Hg3.6*) with relatively high estimated null-allele frequencies in the Baltic and Ladoga samples (see Results). The overall results (not shown) and inferences resulting from the analysis of this 15-locus + mtDNA dataset did not differ from the results of the complete 17-locus + mtDNA dataset, so only the outputs of the main analyses are presented below.

We evaluated the level of confidence in our choice of a best scenario by using simulated pseudo-observed datasets (PODs) to infer the discriminatory power of the analyses (see Cornuet et al. 2010). This was done by simulating 500 PODs for each of the four scenarios by drawing parameter values from their respective prior distributions, and then estimating the relative posterior probability of each competing scenario with respect to each POD, using the same methods and settings as for the real data. Type I error is defined as the probability of the best-fitting scenario *not* having the highest posterior probability when it was in fact used for POD simulation. Conversely, Type II error is the proportion of cases in which the best-fitting scenario was selected although it was not the one used for POD simulation.

## Results

### Genetic diversity within and among subspecies

Consistent genotyping results across all 17 microsatellite loci were obtained for 168 Saimaa, 15 Baltic, and 16 Ladoga ringed seals, while four Saimaa and six Baltic individuals lacked data for a single locus each. No consistent linkage disequilibria across pairs of loci were found in all populations (at *P* < 0.05), indicating absence of structural linkage of markers. Tests for presence of null alleles were significant for six loci in Lake Saimaa (*Hg1.4*, *Hg3.6*, *Hg6.1*, *Hg8.9*, *Hgdii*, and *Pvc78*), but the low estimated frequencies (all *r* < 0.1) suggest that this result simply reflects the presence of population structure within the lake (see Valtonen et al. 2014). Null alleles were also indicated for two loci in the Baltic Sea (*Hg1.4* and *Hg3.6*), and three in Lake Ladoga (*Hg1.4*, *Hg2.3*, and *Hg3.6*); for *Hg1.4* and *Hg3.6*, frequency estimates of these alleles were high at *r* = 0.181–0.286 (Table S3 in Appendix S1).These loci also deviated significantly from HWE due to heterozygote deficiency in all three populations (Table S4 in Appendix S1).

The average number of alleles per locus in the Saimaa population was 3.5, ranging from 2 to 4 in 16 loci, the sole exception being locus *Hl15*, at which 16 alleles were found. In the Baltic and Ladoga populations, the average numbers of alleles per locus were 9.0 and 7.6, respectively, ranging from 4 to 15, even though their sample sizes were an order of magnitude smaller than in Saimaa. Consequently, allelic richness in Saimaa was only about one-third, and expected heterozygosity less than half, of the values observed in the Baltic and Ladoga samples (Table1). Effective inbreeding coefficients, summing the drift within the lacustrine populations in relation to the Baltic, were *F*_e_ = 0.550 for Saimaa, and *F*_e_ = 0.069 for Ladoga.

**Table 1:** Indices of genetic diversity in Baltic, Ladoga, and Saimaa ringed seals based on analyses of 17 microsatellite loci. Numbers of samples (*N*), mean number of alleles (*A*), allelic richness (*A*_R_), observed heterozygosity (*H*_O_), expected heterozygosity (*H*_E_), and inbreeding coefficient (*F*_IS_) are given

Subspecies	*N*	*A* ± SD	*A*_R_	*H*_O_ ± SD	*H*_E_ ± SD	*F*_IS_
Baltic Sea	21	9.00 ± 3.20	8.30	0.74 ± 0.20	0.80 ± 0.08	0.07**
Lake Ladoga	16	7.65 ± 2.76	7.65	0.69 ± 0.22	0.74 ± 0.16	0.06*
Lake Saimaa	172	3.47 ± 3.32	2.77	0.33 ± 0.21	0.36 ± 0.22	0.07***

* *P* < 0.05;

** *P* < 0.01;

*** *P* < 0.001.

As expected (see Chapuis and Estoup 2007), global and pairwise *F*_ST_ estimates based on the original microsatellite dataset were slightly higher than values calculated while correcting for the presence of null alleles. The estimates were nevertheless very similar and, hence, only the values taking the null alleles into account are reported here. Overall differentiation among the subspecies was high (*F*_ST_ = 0.331; 95% CI = 0.247–0.422). Differentiation was statistically significant (*P* < 0.05) across all pairwise comparisons, but markedly higher between Saimaa and the two other populations (*F*_ST(Saimaa–Baltic)_ = 0.367; *F*_ST(Saimaa–Ladoga)_ = 0.343) than between Ladoga and the Baltic Sea (*F*_ST(Ladoga–Baltic)_ = 0.041). For comparison, we note that corresponding indices estimated by Valtonen et al. (2012) based on mtDNA control-region sequences were: overall *F*_ST_ = 0.219; *F*_ST(Saimaa–Baltic)_ = 0.227; *F*_ST(Saimaa–Ladoga)_ = 0.233; and *F*_ST(Ladoga–Baltic)_ = 0.028.

**Figure 4:**
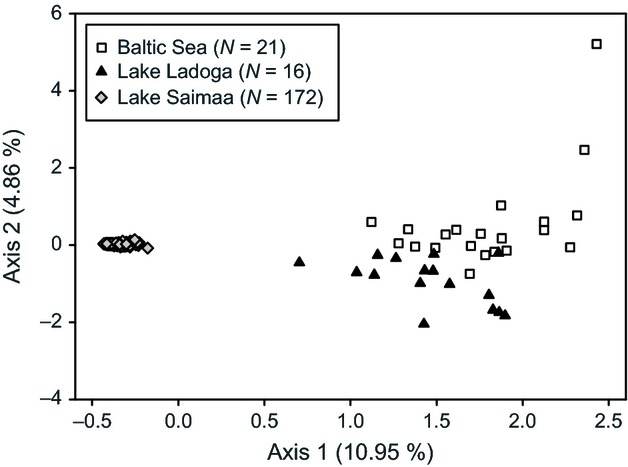
Factorial correspondence analysis (FCA) plot of ringed seal individuals from the Baltic Sea and lakes Saimaa and Ladoga based on their genotypes at 17 microsatellite loci.

In the FCA plot, Saimaa individuals were tightly grouped and clearly separated from the two other subspecies, while Ladoga and Baltic seals formed loose, partially overlapping clusters (Fig.4). Regardless of the model used, Bayesian assignment analyses favored a two-cluster solution for the data (Fig. S1a in Appendix S1; results of the no-admixture runs not shown). The corresponding STRUCTURE assignment plot shows that while Saimaa seals are readily identifiable, individuals from the Baltic and Ladoga cannot be distinguished from each other (Fig. S1b in Appendix S1). Because Kalinowski (2011) has recently shown that the outcome of STRUCTURE analyses can be influenced by unequal sample sizes, we checked the latter result by performing an additional admixture-model run using only Baltic and Ladoga seals. This resulted in highest probability of the data at *K* = 1, maximum Δ*K* at *K* = 4, and ambiguous assignments of all individuals if a four-cluster solution was enforced (results not shown).

### Effective population sizes and divergence times

**Figure 5:**
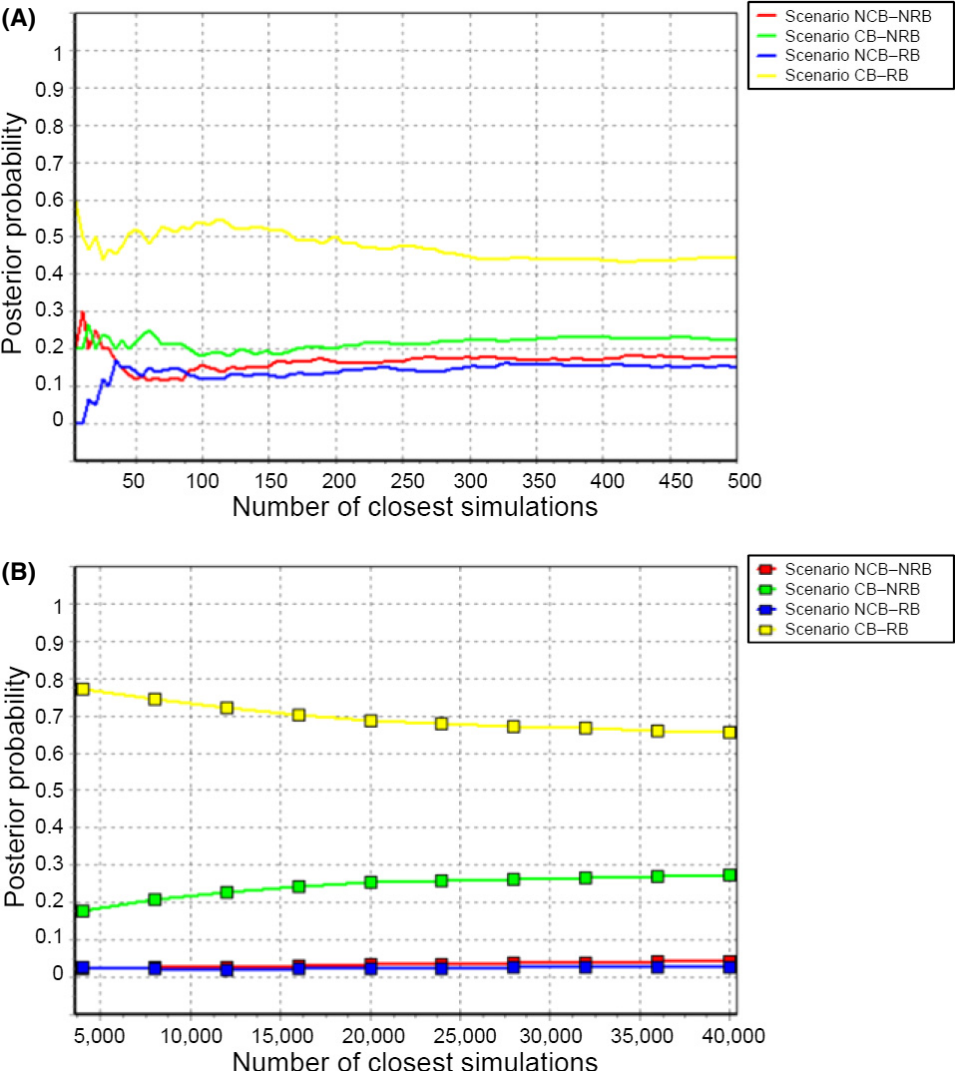
Posterior probabilities of the four alternative hypothetical scenarios based on (a) the direct-estimate approach and (b) the logistic approach.

The direct-estimate comparison of posterior probabilities favored Scenario CB–RB over the simpler alternatives (Fig.5). The superiority of Scenario CB–RB is clearer in the logistic approach, in which the posterior probability of this model at 40,000 closest simulations is 0.66 [95% CI 0.62–0.69]. However, the posterior probability of Scenario CB–NRB is also non-negligible at 0.27 [0.24 –0.30]. As described above, these two scenarios share the assumption of an initial colonization bottleneck in the lacustrine populations, but Scenario CB–RB also invokes a recent bottleneck commencing during the 20th century. By contrast, the posterior probabilities of Scenario NCB–NRB (0.04[0.03–0.06]) and Scenario NCB–RB (0.03[0.02–0.03]), neither of which involves a founder event, are so low that they can safely be rejected as an explanation for the observed data.

Estimations of confidence in scenario choice revealed a fairly high capability of the applied ABC method to discriminate among the scenarios, both when measured as Type I (direct estimate = 0.14; logistic approach = 0.18) and Type II (direct estimate mean = 0.12; logistic approach mean = 0.06) error rates (Table2). However, the mean values of Type II error hide large variation among the three nonoptimal scenarios, because the rates were notably higher than the means when using PODs simulated under Scenario CB–NRB (direct estimate = 0.30; logistic approach = 0.13). By contrast, the probability of making a Type II error was <5% for PODs derived from Scenarios NCB–NRB and NCB–RB (Table2).

**Table 2:** Ability of the applied approximate Bayesian computing (ABC) methods to discriminate among the four hypothetical scenarios, measured as rates of Type I and Type II errors. The latter are shown for all alternative scenarios combined (mean), as well as separately for each suboptimal scenario

Error	Method	

	Direct estimate	Logistic approach
Type I	0.144	0.182
Type II (mean)	0.116	0.055
Type II (Scenario NCB–NRB)	0.010	0.006
Type II (Scenario CB–NRB)	0.296	0.134
Type II (Scenario NCB–RB)	0.042	0.026

NCB–NRB, No Colonization Bottleneck – No Recent Bottleneck.

**Figure 6:**
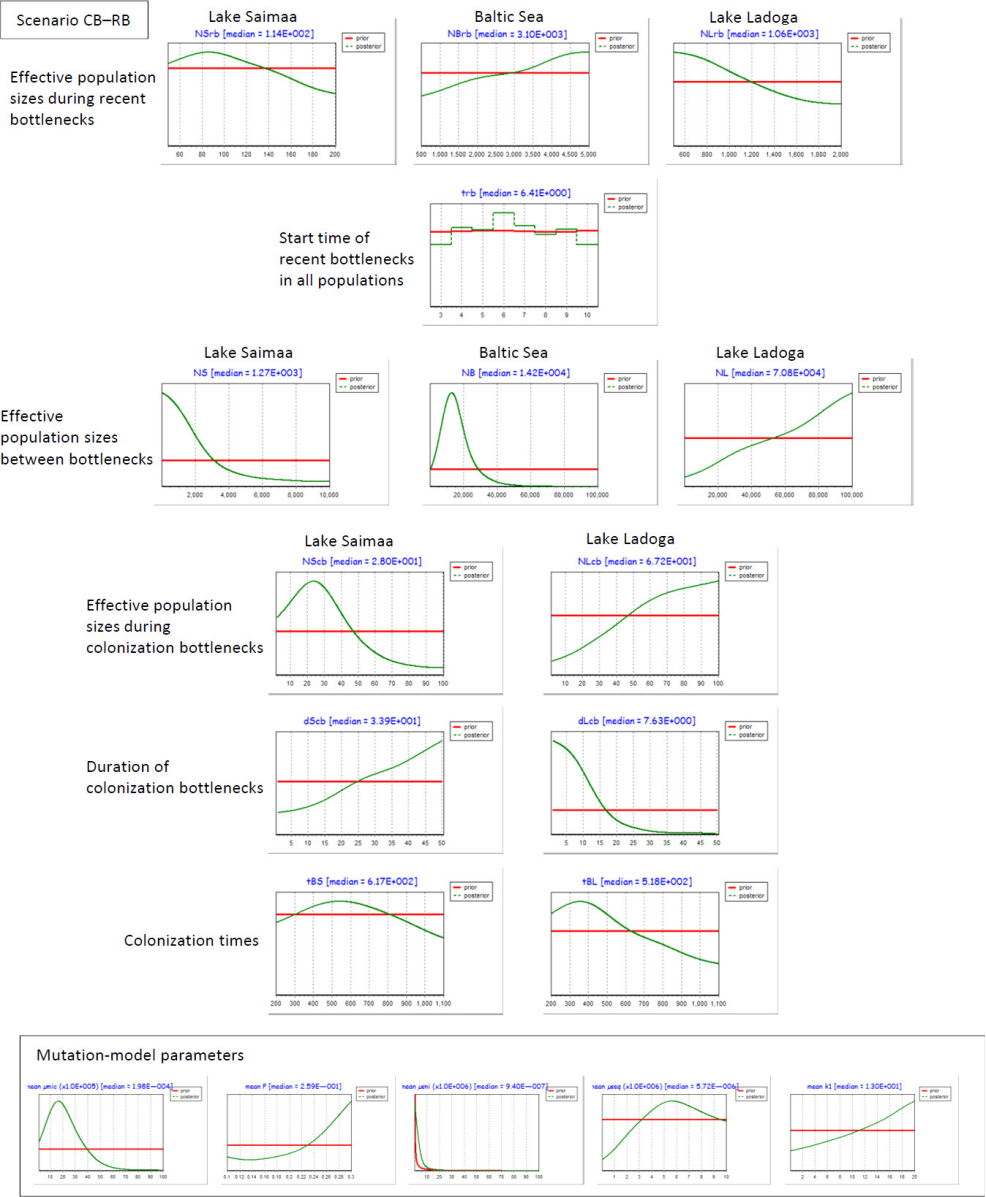
Prior (red lines) and posterior (green lines) distributions of event-time and demographic parameters in three Fennoscandian ringed seal subspecies according to the best-fitting scenario (CB–RB), and estimates of mutation-model parameters for microsatellites and mtDNA sequences (inset).

Posterior distributions of parameter estimates from the best-fitting Scenario CB–RB reveal a rather complex pattern (Fig.6). Estimates for colonization times are equivocal, but the posterior distribution is shifted toward the lower end of the prior range in Lake Ladoga. The results further suggest that post-colonization effective population size within Lake Saimaa remained within a few dozen for tens of generations, while Lake Ladoga appears to have been colonized by a larger number of seals that quickly grew in numbers. In the Baltic population, the long-term median effective size is 14,200 with a 95% CI of [5690–39,100], while corresponding values in Lake Saimaa are 1270 [220–8850] and in Lake Ladoga 70,800 [15,200–98,800]. For Lake Ladoga, however, the posterior distribution is broad. The timing of the recent bottleneck seems uncertain, but the posterior distribution of effective population size tends toward the lower end of the prior range in Saimaa and Ladoga (yet with much higher numbers in the latter), but toward the higher end in the Baltic population (Fig.6).

The second-best hypothesis, Scenario CB–NRB, likewise provides little information on lake colonization times, but also suggests that Saimaa was colonized earlier than Ladoga (Fig. S2 in Appendix S1).The scenario also agrees with Scenario CB–RB with respect to the relative duration and severity of the colonization bottlenecks in the two lakes. The median for effective population size subsequent to the colonization phase in the Baltic Sea is 16,900 [6500 –45,500], in Lake Saimaa 373 [109–2080], and in Lake Ladoga 52,200 [6990–97,600].

For comparison, we note that the simple but disfavored Scenario NCB–NRB, which assumes constant sizes in all populations, gives the following median estimates of long-term effective population sizes: 12,800 [4410–38,500] for the Baltic Sea, 414 [191–736] for Saimaa, and 7810 [1620–70,900] for Ladoga. The likewise disfavored Scenario NCB–RB, which includes only the known recent bottleneck, gives the following pre-bottleneck *N*_e_ estimates: Baltic Sea 10,800 [4000–31,600]; Lake Saimaa 567 [253–1040]; Lake Ladoga 10,500 [1850–73,400]. During the recent bottleneck phase, corresponding values are 3040 [712–4900] for the Baltic Sea, 164 [65–199] for Lake Saimaa, and 1380 [584–1960] for Lake Ladoga, but the posterior distributions are weakly defined especially for the Baltic and Ladoga populations.

Model-checking analyses, in which 200 datasets per favored scenario (CB–RB and CB–NRB) were simulated with parameter values drawn from their respective posterior distributions, revealed that both ‘winning’ scenarios could produce the observed data with high probability; a single outlying summary statistic (at *P* < 0.05) was found for both scenarios when using alternative targets (results not shown). For Scenario CB–RB, this was seen in that the clouds formed by simulated datasets were centered on the observed data in principal components (PCA) space, both when using the original summary statistics (results not shown) and when using an alternative set of target values (Fig. S3 in Appendix S1).

## Discussion

Molecular-genetic surveys of island populations have provided important insights into how small population size, limited gene flow, inbreeding, stochasticity, and isolation time influence the loss of genetic variability in nature (Frankham 1998; Cardoso et al. 2009; Jensen et al. 2013; Wang et al. 2014). The three Fennoscandian ringed seal subspecies constitute an excellent model system for studying such diversity-eroding processes, because the geological history of the Baltic Sea region is well known, and the deglaciation of the area *c*. 10,000 years ago sets a hard upper limit for all divergence times (Ukkonen 2002). Lakes Saimaa and Ladoga were formed in close succession *c*. 9500 years ago (Saarnisto 2011), but their physical features immediately suggest differences in the genetic trajectories of their endemic seal populations: Ladoga is deeper and over four times larger than Saimaa, which is directly reflected in the estimated natural carrying capacities of these lakes (Sipilä et al. 1996; Hyvärinen et al. 1999; Kokko et al. 1999). In addition, the labyrinthine shape of Lake Saimaa has been shown to impose population fragmentation on the seals (Valtonen et al. 2012, 2014), which could speed up genetic erosion in relation to the presumedly more panmictic Ladoga population (*cf*. Amos and Harwood 1998; Caballero et al. 2009). Especially in the case of the Saimaa subspecies, a long-standing issue has been as to whether the majority of the diversity loss can be attributed to a founder effect, long-term erosion, or to the recent bottleneck (Palo et al. 2003).

These kinds of questions were practically intractable until very recently, but the development of coalescent simulation methods, in association with increased computing power, is starting to provide the tools necessary for answering them (Hoban et al. 2012). One of the approaches, approximate Bayesian computing, uses simulated datasets for evaluating how well alternative evolutionary scenarios incorporating differential assumptions concerning (among others) demographic histories explain pre-selected population-genetic ‘target’ parameters (Cornuet et al. 2008; Csilléry et al. 2010). ABC has proven to be a versatile tool that can be used for tackling complex research questions, such as colonization routes of invasive organisms (Boissin et al. 2012; Keller et al. 2012) and hindcasting sizes of endangered (Fontaine et al.^2012^) or even extinct (De Bruyn et al. 2014) populations. In an ABC framework, a clear benefit of the focal Fennoscandian seal populations is that the relative simplicity of the study system makes it possible to formulate distinct alternative demographic hypotheses that can then be tested against each other.

### Genetic diversity within and differentiation among subspecies

As would be expected for a highly mobile circumpolar species with a census size in the millions (Reeves 1998), the ringed seal – as a species – is genetically extremely variable (Palo et al. 2001; O'Corry-Crowe 2008; Olsen et al. 2011; Martinez-Bakker et al. 2013). This species-level diversity does not apply to the three Fennoscandian populations, which have all been more or less isolated from the main Arctic stock since the end of the Pleistocene. The reduction in diversity is relatively mild in the Baltic subspecies, for which previous genetic and ecological modeling analyses suggest connectivity with the Arctic population and a historical census size exceeding 100,000 individuals (Harding and Härkonen 1999; Kokko et al. 1999; Palo et al. 2001). In the scenarios favored by our ABC analyses, the long-term effective size of the Baltic population is estimated to be between 14,000 and 17,000, which is comparable to estimates obtained by Valtonen et al. (2012) in simulations based on mitochondrial control-region sequences (when assuming high mutation rates). In combination, these results therefore indicate that large historical population size – instead of frequent incoming gene flow from the Arctic, as suggested by the microsatellite-based analyses of Martinez-Bakker et al. (2013) – underlies the relatively high genetic diversity in the Baltic Sea. Nevertheless, both our results and the weak Arctic–Baltic differentiation (*F*_ST_ = 0.011–0.028) found in the geographically more extensive survey of Martinez-Bakker et al. (2013) point to the conclusion that postglacial changes in the Baltic population have been relatively minor, meaning that its extant diversity can be assumed to represent reasonably well the initial diversity of the founders of the Saimaa and Ladoga populations.

When it comes to microsatellite intrapopulation diversity and interpopulation differentiation, our results are conveniently summarized in the FCA plot: 172 Saimaa seals form a tight cluster well separated from the others, while, despite the smaller numbers of individuals from the Baltic Sea and Lake Ladoga, high genotypic variability in these populations leads to two wide, partly overlapping ‘clouds’ (Fig.4). Our estimate of a 55% overall loss of heterozygosity in the Saimaa population is slightly lower than the 69% estimate of Palo et al. (2003), but the Saimaa ringed seal still ranks among the genetically most uniform seal populations known (Hoelzel 1999; Gelatt et al. 2010; Han et al. 2010; Schultz et al. 2010; Valtonen et al. 2014; see also O'Corry-Crowe 2008), especially remembering that the reduced nuclear variation is paralleled by losses in mitochondrial haplotype (33.9%) and nucleotide (89.4%) diversity (Valtonen et al. 2012). Prior assessments of nuclear variation are not available for the Ladoga subspecies, but the observed relatively minor reduction (6.9%) in comparison to the Baltic population is in line with the mtDNA results of Valtonen et al. (2012), which showed near-equal haplotype diversities in these large populations (1.0% loss in Ladoga), but also a clear (68.1%) reduction in nucleotide diversity within Lake Ladoga.

### Demographic history of the marine and landlocked subspecies

All Fennoscandian ringed seal populations underwent dramatic, human-caused crashes during the 20th century, but while the Saimaa subspecies only narrowly escaped extinction in the 1980s, the populations of the Baltic Sea and Lake Ladoga remained at (presumably) safer levels (Sipilä et al. 1996; Hyvärinen et al. 1999; Sundqvist et al. 2012; Trukhanova et al. 2013). These events may have contributed substantially to the observed differences in genetic diversity among the populations. However, a viable alternative hypothesis is that variation in the Saimaa population diminished gradually during its long isolation (Palo et al. 2003), or that the population has always been genetically depauperate, which could be the case if it was established by a small number of colonists. Such founder events have been found to underlie the genetic uniformity of many isolated populations (Swatdipong et al. 2010; Uller and Leimu 2011; Kolbe et al. 2012).

In the case of the focal Fennoscandian seals, our ABC results decisively favor the two models assuming founder events in both landlocked populations (Fig.5). However, while posterior distributions suggest an extended initial period of small population size in Lake Saimaa, the colonization bottleneck in Lake Ladoga appears to have been both shorter and less severe (Fig.6 and Fig. S2 in Appendix S1), which could partly explain the preservation of variability in the Ladoga subspecies (*cf*. Clegg et al. 2002; Kekkonen et al. 2012; Keller et al. 2012; see also Valtonen et al. 2012). Posterior distributions of effective population sizes in the lakes during the known recent bottlenecks remain somewhat uncertain, but are shifted toward the lower end of the prior range in both Saimaa and Ladoga (yet with far larger numbers in the latter). The median ABC estimate for Lake Saimaa *N*_e_ during the recent bottleneck (*N*_e_ = 114) is comparable to values estimated by Valtonen et al. (2014) on the basis of linkage disequilibrium (*N*_e_ = 12.3–32.7) and temporal changes in allele frequencies during the last three decades (*N*_e_ = 69–113).This preferred, two-bottleneck interpretation of the history of the Saimaa population exhibits some striking similarities with long-term demographic trends in Black Sea harbor porpoises (*Phocoena phocoena relicta*) estimated recently by Fontaine et al. (2012).

However, it is noteworthy that incorporating the well-known 20th-century bottlenecks into the scenario assuming only lacustrine founder events improves model fit, but not statistically significantly so (Fig.5). Power analyses based on simulated, pseudo-observed datasets also cast doubt on the ability of the applied ABC methods to distinguish between the two best scenarios (Table2). This may indicate that the recent population crashes have not yet had a marked effect, or that they are at least of minor importance compared to the diversity losses incurred by the preceding long isolation of the lacustrine subspecies. Diversity erosion during even severe bottlenecks may be slow (Nei et al. 1975), especially in long-lived species with overlapping generations (Hoffman et al. 2011; Kekkonen et al. 2012). The worst phase of the current bottleneck has lasted as little as five generations, which evidently is not enough to have left a clear signal in the population-genetic parameters of the studied subspecies (see also Schultz et al. 2010; Peery et al. 2012). As a matter of fact, Lander et al. (2011) recently demonstrated that ABC approaches may have a low power to discriminate among scenarios on average, and a low ability to accurately estimate effective population sizes and divergence dates when the temporal scale used is short.

Interestingly, incorporating the recent bottleneck into the founder-event scenario allows a relatively large between-bottleneck population within Lake Saimaa: assuming an *N*_e_/*N*_c_ ratio between 0.1 (Frankham 1995) and 0.16 (Palstra and Ruzzante 2008), the median effective size of 1270 would correspond to survey size of about 8000–13,000 seals. Like the mtDNA-based long-term estimates of Valtonen et al. (2012), these combined-data numbers clearly exceed the estimated carrying capacity of the present-day Lake Saimaa (Hyvärinen et al. 1999), but the estimates can be considered plausible if the wider geological history of the ‘lake district’ of southern Finland is taken into consideration. Due to faster postglacial bedrock rebound along the northern Baltic Sea coastline, the original, northwest-flowing outlets of southern Finnish lakes dried up around 8000 BP (Saarnisto et al. 1999). This damming led to the formation of the Great Lake of Central Finland, an enormous lake complex (Saarnisto et al. 1999; Tikkanen 2002) that could have provided enough space and resources to support up to 10,000 seals (Hyvärinen et al. 1999). When new, southbound rivers finally burst open *c*. 6000 BP onwards, the lake complex slowly became divided into multiple fragments, including Lake Saimaa (Tikkanen 2002; Oinonen et al. 2014). This hypothesis of a wider lacustrine distribution in the past naturally requires that ringed seal populations in the (smaller) lakes adjacent to Saimaa have gone extinct. In addition to the Saimaa area, subfossil seal bones have indeed been recovered from archaeological sites located in the vicinity of the adjacent Lake Päijänne (Ukkonen 2002), but their identification and dating remains disputed (Saarnisto 2011).

Although our ABC analyses provide novel insights into the demographic histories of Fennoscandian ringed seals, they unfortunately leave a few questions open. First, despite the well-established geological chronology of the lakes, the genetic data gave relatively poor estimates for their colonization times within the specified prior range. However, in both favored scenarios, the posterior distributions suggest that Lake Ladoga was colonized later than Saimaa (Fig.6 and Fig. S2 in Appendix S1). Second, posterior distributions of long-term effective population sizes in Lake Ladoga are more or less uninformative in the two models that include founder events (Fig.6 and Fig. S2 in Appendix S1). This could indicate that enforcing a hypothesis of a small colonizing population in the case of Ladoga necessitates overcompensation in the simulated long-term effective population sizes. While the disfavored scenarios NCB–NRB and NCB–RB produced more focused posteriors within Ladoga (Fig. S4 in Appendix S1), median *N*_e_ estimates in these are still only slightly lower than the Baltic numbers and, hence, when converted to ‘real’ seals, are too high even for such a large lake. Especially in the case of the more complex scenarios, these ambiguities are almost certainly partly brought about by the relatively small Ladogan sample size (*cf*. Hale et al. 2012), but we also propose that this could be caused by a violation of one of the central assumptions in simulations in DIYABC, namely, the absence of gene flow after population separation (Cornuet et al. 2010; Lander et al. 2011). After its initial formation, Lake Ladoga remained only a few meters above sea level and broadly connected with the Baltic basin during at least some parts of the Litorina Sea stage, *c*. 7500–4000 BP (Ukkonen 2002; Saarnisto 2011). Even today, the elevation difference between Lake Ladoga and the Baltic Sea is only five meters, meaning that marine seals could conceivably reach the lake via its 70 km long current outlet, river Neva (Saarnisto 2011). Ladoga seals are morphologically differentiated from the Baltic subspecies (Sipilä et al. 1996), and the general genetic compositions of the populations clearly differ (Fig.4), but it is noteworthy that Bayesian assignment analyses based on presumably neutral microsatellites have difficulties with identifying the source population of Baltic or Ladogan individuals (Fig. S1 in Appendix S1).

## Conclusions

Our ABC simulations shed light on the origin of the widely differing levels of nuclear and mitochondrial diversity in the three Fennoscandian ringed seal subspecies. The results strongly suggest that Lake Saimaa was colonized by a small number of seals, and that a significant portion of the genetic variation present in the marine colonizers was lost already during this prolonged founder event, with slow further erosion taking place throughout the post-colonization period. The ensuing low variability evidently has not prevented the Saimaa population from persisting for nearly ten thousand years, a possibility that is supported by numerous examples of long-term survival of genetically homogeneous species (Schultz et al. 2010; Rodríguez et al. 2011) and even rapid population growth under suitable conditions (Hoelzel 1999; Taylor et al. 2005; Bouzat 2010; Rollins et al. 2013). This indicates that lack of heritable variation per se would not prevent recovery of the Saimaa population, if current anthropogenic threats could be controlled effectively. The best-fitting scenario also includes the known 20th-century population crash in all subspecies, but the effect is weak, indicating that the bottleneck is too recent to manifest clearly in the genetic composition of the populations. However, we note that Valtonen et al. (2014) found evidence for a slow (yet statistically significant) decline in individual heterozygosity within the Saimaa population during the last five decades; preventing further diversity losses in this critically endangered subspecies therefore most likely requires increasing its head count.

Ladoga ringed seals present a more complicated case, because the population is genetically nearly as diverse as the Baltic subspecies in both nuclear microsatellites (this study) and mitochondrial control-region sequences (Valtonen et al. 2012). Our simulations indicate that the lake was colonized by a large number of seals, and that the initial bottleneck – if it can be called such – was brief. Although lake colonization times cannot be inferred with confidence based on the present data, all evaluated scenarios indicate that that the final separation of the Ladoga population occurred later than in Lake Saimaa, which is in line with the prevailing view of the geological history of these large lake basins (Ukkonen 2002; Saarnisto 2011). Estimating the size of the Ladoga population likewise proved to be problematic: posterior distributions in the favored scenarios are quite uninformative, while the disfavored scenarios produce numbers close to the Baltic estimates. Such large populations cannot be supported by the lake, but the results could reflect intermittent incoming gene flow from the Baltic Sea. Future studies of Ladoga ringed seals, employing larger numbers of markers and individuals, are clearly warranted.

## Data Accessibility

Microsatellite genotypes and mtDNA sequences have been deposited as a DIYABC-formatted text file in the DRYAD database, along with the reftableHeaders file containing the scenarios, priors, and settings used in the main analyses (doi:10.5061/dryad.r25g1).

## Supporting Information

**Appendix S1.** Supplementary tables and figures. **Table S1.** Mutation-model priors used for microsatellite loci and mitochondrial control-region sequences in the ABC analyses. **Table S2.** Target summary statistics used during ABC simulations and model-checking analyses. **Table S3.** Observed and estimated microsatellite allele frequencies in the three ringed seal subspecies. **Table S4.** Sample sizes, diversity indices, estimated null-allele frequencies, and deviations from Hardy–Weinberg equilibrium at 17 microsatellite loci in the focal ringed seal subspecies. **Figure S1.** (a) Δ*K* values for each number of *K* from STRUCTURE runs, and (b) assignment of Saimaa, Ladoga, and Baltic ringed seal individuals into two population clusters in STRUCTURE. **Figure S2.** Prior (red lines) and posterior (green lines) distributions of event-time and demographic parameters in three Fennoscandian ringed seal subspecies according to the second-best scenario (CB–NRB), and estimates of mutation-model parameters for microsatellites and mtDNA sequences (inset). **Figure S3.** PCA plots from a model-checking analysis based on alternative target statistics and datasets simulated using parameter values drawn from the posterior distributions from Scenario CB–RB. **Figure S4.** Prior (red lines) and posterior (green lines) distributions for effective population size in Ladoga ringed seals in each of the four scenarios evaluated in the ABC analyses.
