# Supplementary material for: Demographic histories and genetic diversities of Fennoscandian marine and landlocked ringed seal subspecies
Source: Ecol Evol. 2014 Aug 19;4(17):3420–34. doi: 10.1002/ece3.1193 (PMC4228616; doi:10.1002/ece3.1193)
Supplement: Supplementary file 1 [file ece30004-3420-sd1.pdf]

Supporting Information (Nyman *et al.*)

Appendix S1. Supplementary tables and figures.

Table S1 Mutation-model priors used for microsatellite loci and mitochondrial control-region sequences in the ABC analyses

Marker, parameter	Interpretation	Distribution	Minimum	Maximum	Mean	Shape parameter
**Microsatellites**						
μ	Mean mutation rate	Uniform	1E-5	1E-3		
μi	Individual locus mutation rate	Gamma	1E-6	1E-2	μ	2
P	Mean parameter of the geometric distribution of the change in number of repeats at mutation events	Uniform	0.1	0.3		
Pi	Individual locus P	Gamma	0.01	0.9	P	2
μSNI	Mean single nucleotide insertion/deletion rate	Log-uniform	1E-8	1E-4		
μSNIi	Individual locus single nucleotide insertion/deletion rate	Gamma	1E-9	1E-3	μSNI	2
**mtDNA control-region sequences (substitution model = HKY+I[88%]+G[1.40])**						
μSEQ	Mean mutation rate per site per generation	Uniform	1E-8	1E-5		
μSEQi	Individual locus mutation rate (not needed)	Uniform	1E-9	1E-4	μSEQ	2
k	Mean coefficient kappa (transition-transversion bias)	Uniform	0.05	20		
ki	Individual locus coefficient kappa (not needed)	Uniform	0.05	20	k	2

Table S2 Target summary statistics used during ABC simulations and model-checking analyses

Loci and summary statistics	Simulations	Model checking
**Microsatellite loci**		
**One-sample summary statistics**		
Mean number of alleles	**x**	
Mean genic diversity (expected heterozygosity)	**x**	
Mean allele size variance		**x**
Mean Garza-Willamson's *M*	**x**	
**Two-sample summary statistics**		
Mean number of alleles	**x**	
Mean genic diversity (expected heterozygosity)		**x**
Mean allele size variance		**x**
*F*st	**x**	
Classification index (assignment likelihood)	**x**	
Shared allele distance		**x**
(du)2 distance		**x**
**Mitochondrial control-region sequences**		
**One-sample summary statistics**		
Number of haplotypes	**x**	
Number of segregating sites	**x**	
Mean of pairwise differences	**x**	
Variance of pairwise differences	**x**	
Tajima's *D*		**x**
Private segregating sites		**x**
Mean of numbers of the rarest nucleotide at segregating sites		**x**
**Two-sample summary statistics**		
Number of haplotypes	**x**	
Number of segregating sites	**x**	
Mean of pairwise differences (W)		**x**
Mean of pairwise differences (B)		**x**
*F*st	**x**	

Table S3 Observed and estimated microsatellite allele frequencies in ringed seals from Lake Saimaa ($N = 172$), the Baltic Sea ($N = 21$), and Lake Ladoga ($N = 16$)

		Lake Saimaa		Baltic Sea		Lake Ladoga	
Locus	Allele size	Observed	Estimated	Observed	Estimated	Observed	Estimated
Hg1.4	185	0.012	0.009	0.175	0.112	–	–
	186	–	–	–	–	0.063	0.063
	187	–	–	0.600	0.445	0.219	0.171
	188	–	–	–	–	0.188	0.109
	189	–	–	0.100	0.054	0.281	0.214
	190	–	–	–	–	0.063	0.033
	191	0.988	0.942	0.075	0.052	0.156	0.104
	193	–	–	0.025	0.025	–	–
	195	–	–	–	–	0.031	0.031
	197	–	–	0.025	0.025	–	–
	0	–	0.049	–	0.286	–	0.276
Hg2.3	110	–	–	0.143	0.133	0.031	0.031
	114	0.172	0.167	0.071	0.071	0.219	0.187
	116	–	–	0.071	0.071	0.063	0.063
	118	–	–	0.190	0.174	–	–
	120	–	–	0.048	0.048	0.156	0.138
	122	–	–	0.095	0.095	0.063	0.063
	124	0.828	0.808	0.143	0.122	0.375	0.329
	126	–	–	–	–	0.063	0.063
	128	–	–	0.119	0.119	–	–
	130	–	–	0.048	0.048	–	–
	132	–	–	0.024	0.024	0.031	0.031
	134	–	–	0.024	0.024	–	–
	136	–	–	0.024	0.024	–	–
	0	–	0.025	–	0.048	–	0.096
Hg3.6	94	–	–	0.111	0.063	–	–
	96	–	–	0.444	0.369	0.094	0.067
	98	0.061	0.061	0.056	0.056	0.063	0.063
	100	0.366	0.346	0.139	0.117	0.125	0.125
	102	–	–	0.139	0.093	0.094	0.067
	104	0.573	0.546	0.028	0.028	0.063	0.034
	106	–	–	–	–	0.031	0.031
	108	–	–	0.028	0.028	0.531	0.431
	110	–	–	0.056	0.030	–	–
	0	–	0.047	–	0.217	–	0.181
Hg4.2	132	–	–	–	–	0.094	0.094
	134	0.384	0.368	0.286	0.286	0.344	0.344
	140	–	–	0.071	0.071	0.031	0.031
	142	–	–	0.024	0.024	0.063	0.063
	144	–	–	0.048	0.048	–	–
	148	–	–	0.048	0.048	0.031	0.031
	150	–	–	0.048	0.048	0.063	0.063
	152	–	–	0.048	0.048	–	–
	154	–	–	0.214	0.214	0.063	0.063
	156	–	–	0.071	0.071	0.281	0.281
	157	–	–	–	–	0.031	0.031
	160	–	–	0.095	0.095	–	–
	161	0.549	0.523	–	–	–	–
	163	0.067	0.055	–	–	–	–
	165	–	–	0.024	0.024	–	–
	169	–	–	0.024	0.024	–	–
	0	–	0.053	–	0.000	–	0.000
Hg6.1	146	–	–	0.024	0.024	0.063	0.063
	148	0.023	0.021	0.024	0.024	–	–
	150	–	–	–	–	0.125	0.125
	152	0.977	0.940	0.310	0.310	0.375	0.375
	154	–	–	0.024	0.024	–	–
	156	–	–	–	–	0.031	0.031
	158	–	–	0.119	0.119	0.063	0.063
	160	–	–	0.048	0.048	0.063	0.063
	162	–	–	0.119	0.119	–	–
	164	–	–	–	–	0.063	0.063
	166	–	–	0.048	0.048	–	–
	167	–	–	0.119	0.119	–	–
	171	–	–	0.024	0.024	0.031	0.031
	173	–	–	0.024	0.024	0.094	0.094
	174	–	–	0.048	0.048	–	–
	176	–	–	0.024	0.024	–	–
	178	–	–	0.048	0.048	0.063	0.063
	180	–	–	–	–	0.031	0.031
	0	–	0.040	–	0.000	–	0.000
Hg8.9	188	–	–	–	–	0.156	0.156
	190	–	–	0.071	0.071	0.219	0.219
	192	–	–	0.095	0.095	0.031	0.031
	194	0.073	0.069	0.381	0.381	0.219	0.219
	196	–	–	0.119	0.119	0.063	0.063
	198	–	–	–	–	0.063	0.063
	200	0.372	0.345	0.024	0.024	0.063	0.063
	202	0.555	0.517	0.048	0.048	0.031	0.031
	204	–	–	0.143	0.143	0.125	0.125
	206	–	–	0.095	0.095	–	–
	208	–	–	0.024	0.024	0.031	0.031
	0	–	0.069	–	0.000	–	0.000
Hg8.10	180	–	–	0.025	0.025	–	–
	184	–	–	0.075	0.075	0.031	0.031
	186	0.828	0.828	0.450	0.450	0.625	0.625
	188	0.172	0.172	0.350	0.350	0.281	0.281
	190	–	–	–	–	0.063	0.063
	192	–	–	0.050	0.050	–	–
	194	–	–	0.050	0.050	–	–
	0	–	0.000	–	0.000	–	0.000
Hgdii	200	0.924	0.869	0.167	0.167	0.313	0.306
	206	–	–	0.024	0.024	–	–
	208	–	–	0.095	0.095	0.094	0.094
	214	–	–	0.119	0.119	0.031	0.031
	216	–	–	0.119	0.119	–	–
	218	–	–	0.357	0.357	0.219	0.219
	220	0.009	0.009	0.095	0.095	0.156	0.151
	222	–	–	0.024	0.024	0.188	0.183
	228	0.067	0.059	–	–	–	–
	0	–	0.064	–	0.000	–	0.016

Table S3 (Continued)

		Lake Saimaa		Baltic Sea		Lake Ladoga	
Locus	Allele size	Observed	Estimated	Observed	Estimated	Observed	Estimated
Hl15	129	0.070	0.069	0.024	0.024	0.156	0.156
	131	–	–	0.024	0.024	–	–
	133	0.003	0.003	0.024	0.024	–	–
	135	0.006	0.006	0.024	0.024	–	–
	137	0.488	0.481	0.024	0.024	–	–
	139	0.026	0.026	–	–	–	–
	141	–	–	–	–	0.031	0.031
	143	–	–	–	–	0.031	0.031
	145	–	–	0.071	0.071	0.031	0.031
	147	–	–	–	–	0.063	0.063
	149	–	–	0.119	0.119	–	–
	151	0.015	0.015	0.048	0.048	0.063	0.063
	153	0.070	0.070	0.119	0.119	0.125	0.125
	155	0.061	0.061	0.167	0.167	0.125	0.125
	157	0.015	0.015	0.119	0.119	0.094	0.094
	159	0.067	0.065	0.071	0.071	0.063	0.063
	161	0.012	0.012	0.119	0.119	0.063	0.063
	163	0.006	0.006	0.024	0.024	0.063	0.063
	165	0.061	0.061	–	–	–	–
	167	0.079	0.079	0.024	0.024	0.031	0.031
	169	0.018	0.016	–	–	0.031	0.031
	171	0.003	0.003	–	–	0.031	0.031
	0	–	0.012	–	0.000	–	0.000
Pvc19	104	–	–	0.024	0.024	–	–
	106	–	–	0.024	0.024	0.031	0.031
	108	0.105	0.104	0.071	0.071	–	–
	110	–	–	0.238	0.238	0.219	0.219
	112	0.221	0.217	0.048	0.048	0.031	0.031
	114	–	–	0.024	0.024	0.031	0.031
	116	0.006	0.006	0.048	0.048	0.063	0.063
	118	0.669	0.660	0.405	0.405	0.531	0.531
	120	–	–	0.071	0.071	0.094	0.094
	122	–	–	0.048	0.048	–	–
	0	–	0.013	–	0.000	–	0.000
Pvc26	103	0.003	0.003	0.214	0.214	0.031	0.031
	105	0.009	0.006	0.310	0.309	0.500	0.500
	107	–	–	0.381	0.381	0.406	0.406
	109	0.866	0.843	0.095	0.095	0.063	0.063
	111	0.123	0.121	–	–	–	–
	0	–	0.027	–	0.001	–	0.000
Pvc30	167	–	–	–	–	0.031	0.031
	171	0.347	0.344	0.238	0.238	0.219	0.212
	173	–	–	–	–	0.063	0.063
	177	0.065	0.065	–	–	0.063	0.063
	179	0.106	0.105	0.238	0.238	0.125	0.125
	181	0.482	0.479	0.143	0.143	–	–
	183	–	–	0.024	0.024	–	–
	185	–	–	0.238	0.238	0.344	0.334
	187	–	–	0.095	0.095	0.094	0.081
	189	–	–	0.024	0.024	0.063	0.063
	0	–	0.007	–	0.000	–	0.029
Pvc78	137	–	–	0.400	0.400	0.094	0.094
	139	0.172	0.153	0.375	0.375	0.500	0.500
	141	–	–	0.025	0.025	0.125	0.125
	143	–	–	0.075	0.075	0.031	0.031
	145	–	–	0.075	0.075	–	–
	147	–	–	0.050	0.050	0.031	0.031
	149	0.828	0.764	–	–	0.219	0.219
	0	–	0.083	–	0.000	–	0.000
SGPv9	161	0.904	0.904	0.429	0.422	0.875	0.875
	163	–	–	0.024	0.024	–	–
	165	–	–	0.024	0.024	0.031	0.031
	167	–	–	0.071	0.071	0.031	0.031
	169	–	–	–	–	0.031	0.031
	171	0.096	0.096	0.238	0.235	0.031	0.031
	173	–	–	0.214	0.208	–	–
	0	–	0.000	–	0.015	–	0.000
SGPv10	131	–	–	0.024	0.024	–	–
	133	0.317	0.317	–	–	–	–
	135	–	–	0.024	0.024	0.094	0.094
	137	–	–	0.071	0.071	–	–
	139	–	–	0.024	0.024	0.156	0.156
	141	–	–	0.310	0.295	0.313	0.310
	143	–	–	0.119	0.119	0.063	0.063
	145	0.683	0.683	0.024	0.024	0.094	0.094
	147	–	–	0.214	0.208	0.063	0.053
	149	–	–	–	–	0.031	0.031
	151	–	–	0.024	0.024	0.063	0.063
	153	–	–	0.119	0.119	0.094	0.094
	155	–	–	0.048	0.031	–	–
	157	–	–	–	–	0.031	0.031
	0	–	0.000	–	0.038	–	0.012
SGPv11	152	–	–	0.071	0.071	0.063	0.063
	154	–	–	0.167	0.167	0.375	0.375
	156	0.503	0.503	0.310	0.310	0.375	0.375
	157	0.331	0.331	–	–	–	–
	158	–	–	0.119	0.119	0.063	0.063
	159	–	–	0.071	0.071	–	–
	160	–	–	0.024	0.024	–	–
	162	–	–	0.071	0.071	–	–
	169	0.166	0.166	0.048	0.048	0.031	0.031
	174	–	–	0.048	0.048	0.031	0.031
	177	–	–	0.024	0.024	0.063	0.063
	179	–	–	0.024	0.024	–	–
	183	–	–	0.024	0.024	–	–
	0	–	0.000	–	0.000	–	0.000
SGPv16	117	–	–	0.238	0.238	0.438	0.438
	119	0.061	0.060	0.119	0.117	0.188	0.188
	121	–	–	0.048	0.048	0.063	0.063
	123	0.939	0.929	0.238	0.236	0.125	0.125
	125	–	–	0.190	0.189	0.031	0.031
	127	–	–	0.024	0.024	–	–
	133	–	–	0.143	0.143	0.156	0.156
	0	–	0.011	–	0.005	–	0.000

Table S4 Genetic diversity of the Baltic, Ladoga, and Saimaa ringed seal subspecies at 17 microsatellite loci: N = sample size, A = number of alleles, r = estimated null allele frequency, H_O = observed heterozygosity, H_E = expected heterozygosity; (uncorrected) P-values in bold indicate significant deviations from Hardy-Weinberg equilibrium

	Locus																	
Population	*Hg1.4*	*Hg2.3*	*Hg3.6*	*Hg4.2*	*Hg6.1*	*Hg8.9*	*Hg8.10*	*Hgdii*	*Hl15*	*Pvc19*	*Pvc26*	*Pvc30*	*Pvc78*	*SGPv9*	*SGPv10*	*SGPv11*	*SGPv16*	Mean
Baltic Sea																		
N	20	21	18	21	21	21	20	21	21	21	21	21	20	21	21	21	21	
A	6	12	8	12	14	9	6	8	15	10	4	7	6	6	11	12	7	9.000
r	0.286	0.048	0.217	0.000	0.000	0.000	0.000	0.000	0.000	0.000	0.001	0.000	0.000	0.015	0.038	0.000	0.005	0.036
H_O	0.150	0.762	0.389	0.857	0.905	0.857	0.800	0.810	0.952	0.762	0.667	0.857	0.750	0.667	0.762	0.857	0.810	0.742
H_E	0.608	0.905	0.765	0.863	0.870	0.813	0.681	0.816	0.921	0.779	0.721	0.819	0.703	0.725	0.840	0.861	0.833	0.795
P	**0.000**	0.104	**0.000**	0.314	0.930	0.313	0.367	0.724	0.824	0.636	0.274	0.450	1.000	0.881	0.180	0.860	0.266	
Lake Ladoga																		
N	16	16	16	16	16	16	16	16	16	16	16	16	16	16	16	16	16	
A	7	8	7	9	11	10	4	6	15	7	4	8	6	5	10	7	6	7.647
r	0.276	0.096	0.181	0.000	0.000	0.000	0.000	0.016	0.005	0.000	0.000	0.029	0.000	0.000	0.012	0.001	0.000	0.036
H_O	0.313	0.563	0.375	0.875	0.813	1.000	0.563	0.750	0.875	0.813	0.688	0.750	0.750	0.250	0.875	0.625	0.875	0.691
H_E	0.831	0.798	0.698	0.804	0.839	0.877	0.542	0.810	0.940	0.675	0.599	0.823	0.698	0.238	0.865	0.728	0.752	0.736
P	**0.000**	0.066	**0.001**	0.987	0.452	0.810	1.000	0.656	**0.017**	0.646	0.899	0.235	0.702	1.000	0.240	0.910	0.170	
Lake Saimaa																		
N	172	172	172	172	172	172	172	172	171	172	171	170	172	172	172	172	172	
A	2	2	3	3	2	3	2	3	16	4	4	4	2	2	2	3	2	3.471
r	0.049	0.025	0.047	0.053	0.040	0.069	0.000	0.064	0.012	0.013	0.027	0.007	0.083	0.000	0.000	0.000	0.011	0.029
H_O	0.012	0.262	0.448	0.517	0.035	0.436	0.297	0.105	0.673	0.477	0.234	0.618	0.203	0.192	0.459	0.599	0.110	0.334
H_E	0.023	0.285	0.536	0.548	0.046	0.550	0.285	0.141	0.734	0.495	0.236	0.633	0.285	0.174	0.434	0.612	0.115	0.361
P	**0.018**	0.287	**0.006**	**0.000**	0.080	**0.008**	0.789	**0.007**	**0.006**	0.180	**0.033**	0.240	**0.001**	0.370	0.483	0.637	0.477	

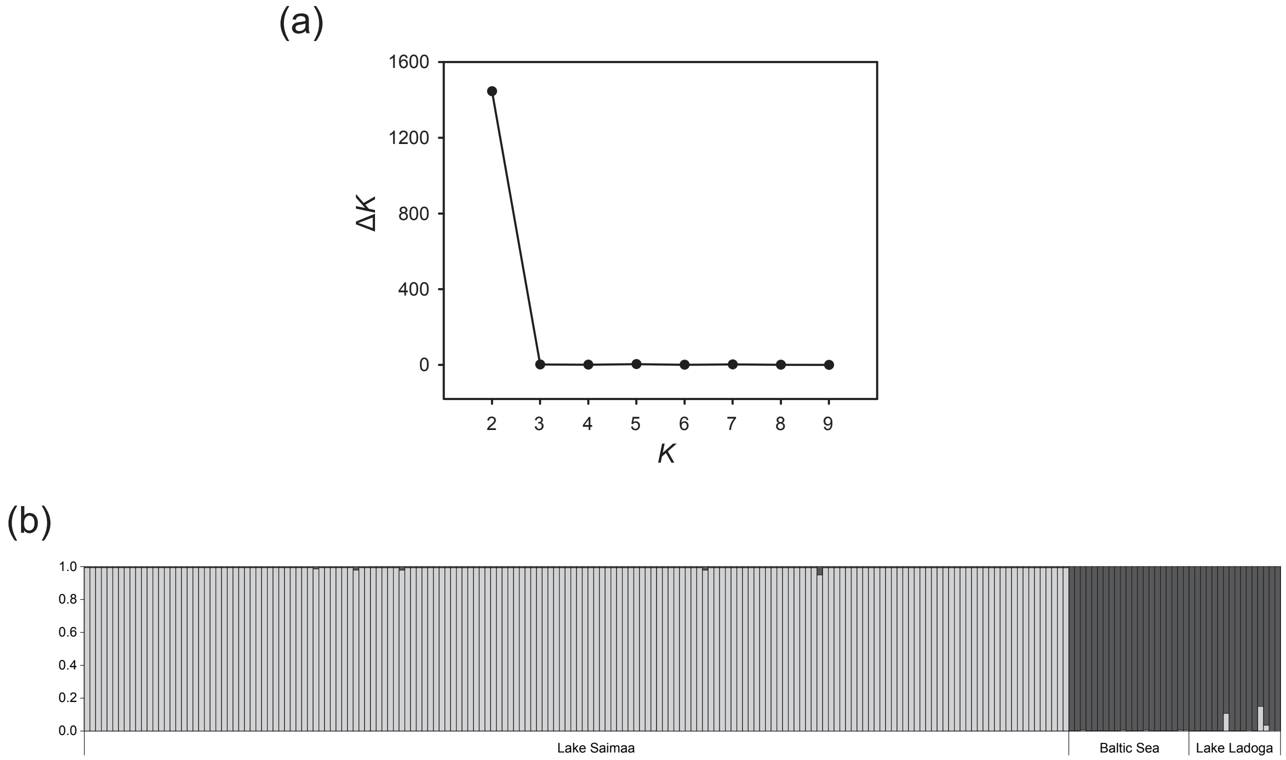

Fig. S1 (a) ΔK values for each number of K from STRUCTURE runs, and (b) assignment of Saimaa, Ladoga, and Baltic ringed seal individuals into two population clusters in STRUCTURE.

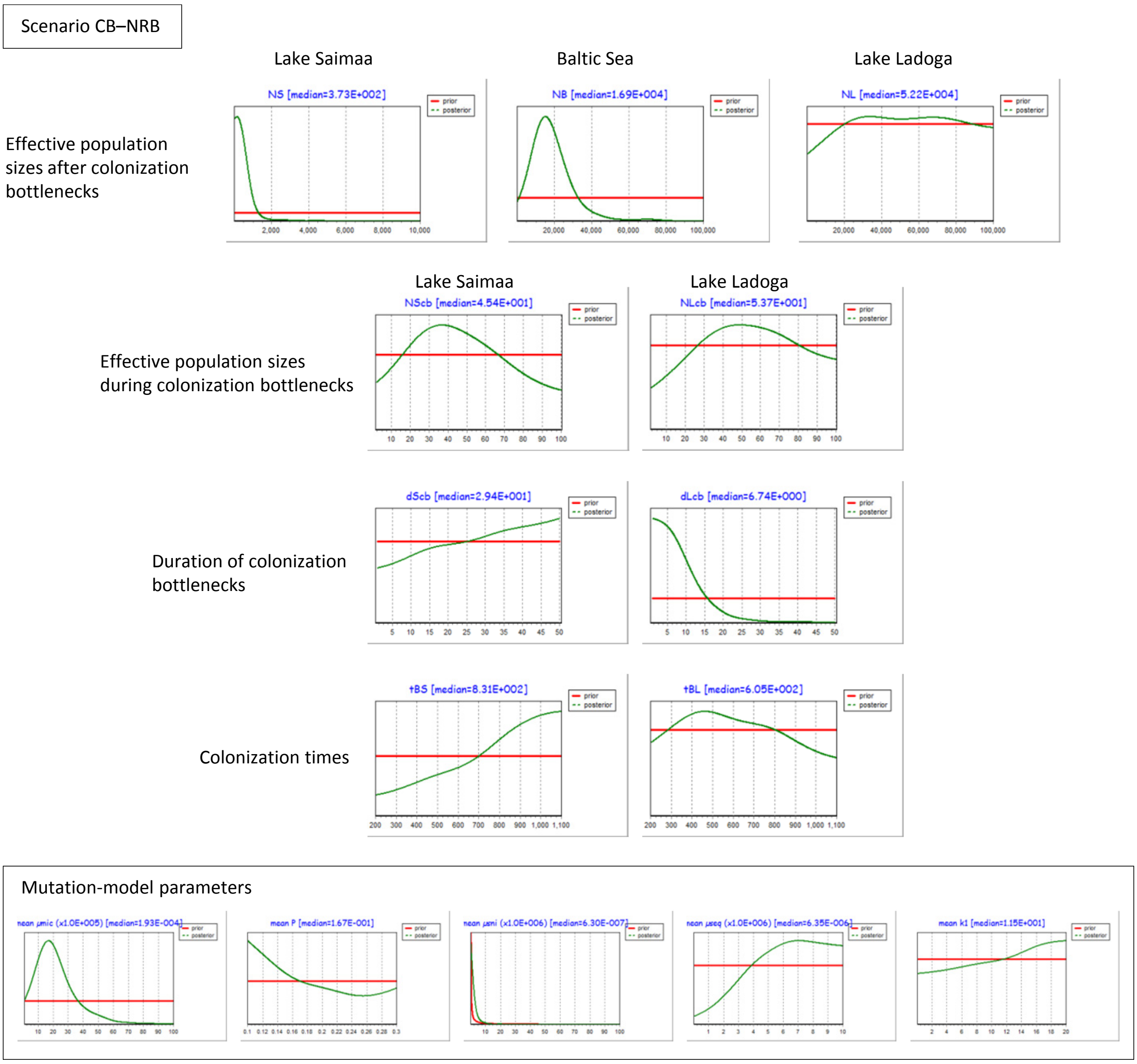

Fig. S2 Prior (red lines) and posterior (green lines) distributions of event-time and demographic parameters in three Fennoscandian ringed seal subspecies according to the second-best scenario (CB–NRB), and estimates of mutation-model parameters for microsatellites and mtDNA sequences (inset).

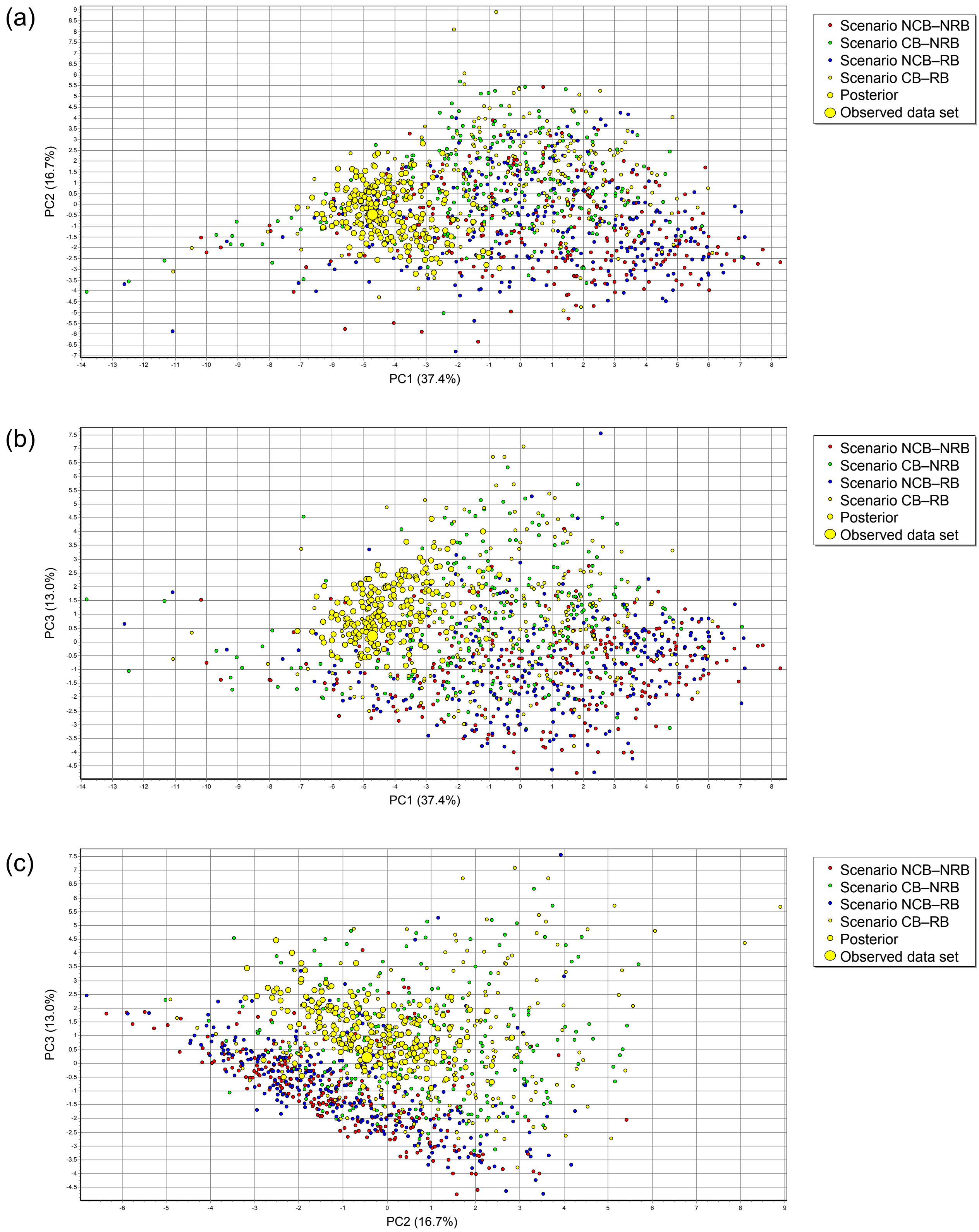

Fig. S3 PCA plots (first three axes) from a model-checking analysis based on alternative target statistics and datasets simulated using parameter values drawn from the posterior distributions from Scenario CB–RB.

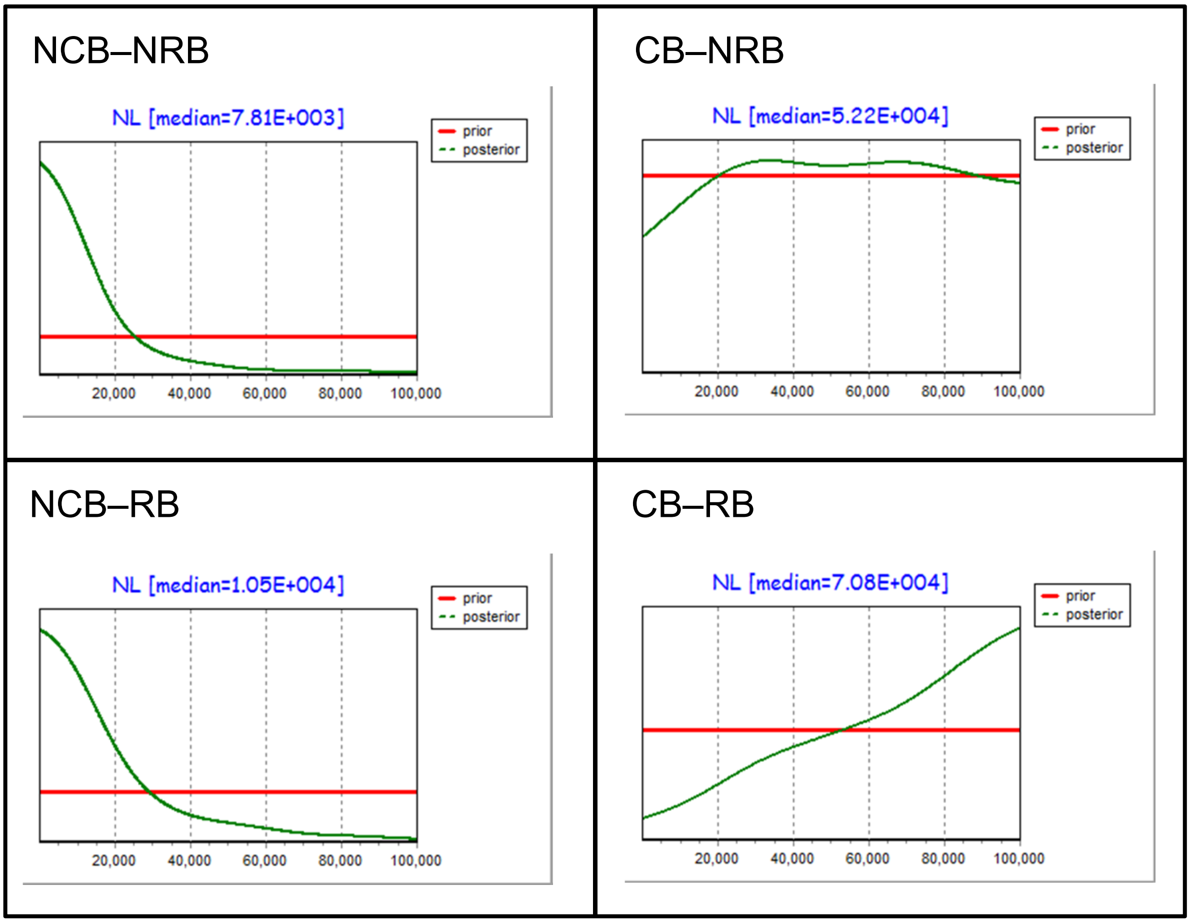

Fig. S4 Prior (red lines) and posterior (green lines) distributions for effective population size in Ladoga ringed seals in each of the four scenarios evaluated in the ABC analyses.
